# Clinical Characteristics of Infants Under Two Years of Age with Early Sensitization to House Dust Mites

**DOI:** 10.3390/jcm14186587

**Published:** 2025-09-18

**Authors:** Hye-In Jeong, You Hoon Jeon

**Affiliations:** Department of Pediatrics, Hallym University Dongtan Sacred Heart Hospital, Hwaseong 18450, Republic of Korea; hyein0613@hallym.or.kr

**Keywords:** allergens, house dust mites, immunoglobulin E, infants

## Abstract

**Background:** Early-life sensitization to house dust mites (HDMs) is a recognized risk factor for adverse respiratory allergic outcomes. **Methods:** We investigated the clinical characteristics of infants under two years of age who visited our allergy clinic for evaluation with detectable HDM-specific IgE (sIgE) and compared them to HDM-sIgE–negative infants. **Results:** Among 1793 infants tested for HDM sIgE, 96 (5.4%) demonstrated sensitization. In the HDM-positive cohort, the prevalence of atopic dermatitis was 74.0% (90.9% among those <12 months), food allergy was 57.3% (100% among those <12 months), egg white sensitization was 71.9% (90.9% among those <12 months), and cow’s milk sensitization was 56.3% (81.8% among those <12 months). Atopic dermatitis, food allergy, ≥4 wheezing episodes, physician-diagnosed asthma, allergic rhinitis, egg white sensitization, cow’s milk sensitization, and sensitization to three or more food allergens were significantly more common in the HDM-positive group compared with the HDM-negative group. Significant correlations were observed between HDM sIgE and total IgE levels, as well as between HDM sIgE and egg white sIgE levels. Overall, HDM sensitization in infants was most frequently accompanied by atopic dermatitis and egg white sensitization. **Conclusions:** These findings suggest that early HDM sensitization should be closely monitored, particularly in infants with atopic dermatitis and food allergies who exhibit elevated total IgE and egg white sIgE levels.

## 1. Introduction

House dust mites (HDMs) have long been recognized as clinically relevant indoor allergens and remain among the most important triggers of allergic disease worldwide [1,2]. Among indoor allergens, HDMs are the most prevalent triggers of allergic sensitization, particularly in respiratory disorders. Two species, *Dermatophagoides farinae* (*D. farinae*) and *Dermatophagoides pteronyssinus* (*D. pteronyssinus*), collectively account for approximately 90% of the global HDM population. The relative distribution of these species varies geographically. In Korea, HDMs are predominantly composed of *D. farinae* (approximately 65%), followed by *D. pteronyssinus* (around 21%) [3]. Correspondingly, sensitization to *D. farinae* is generally more frequent than to *D. pteronyssinus*, with this disparity being particularly pronounced in pediatric populations, where sensitization to *D. farinae* typically precedes that to *D. pteronyssinus* [4,5,6].

Pediatric allergic diseases frequently progress in a sequence commonly referred to as the “allergic march.” This process usually begins with atopic dermatitis, followed by food sensitization and clinical food allergy, and later inhalant allergen sensitization that contributes to respiratory conditions such as rhinitis and asthma. As children reach preschool age, sensitization to inhalant allergens, particularly HDMs, becomes more prominent than sensitization to food allergens and frequently leads to respiratory allergic diseases, including allergic rhinitis and asthma [7,8,9]. In Korea, the prevalence of HDM sensitization among patients with atopic dermatitis has been reported to range from 27.9% to 68.8%, while 40% to 60% of patients with respiratory allergies, allergic rhinitis, or asthma demonstrate HDM sensitivity [3]. More than half of asthmatic patients are sensitized to HDMs, and both the incidence and severity of asthma are strongly associated with HDM allergy [10,11,12].

Early-life exposure to HDMs plays a pivotal role in the development of HDM sensitization and infantile asthma [13,14]. Furthermore, early HDM sensitization has been linked to impaired lung function and is considered a significant risk factor for unfavorable respiratory allergic outcomes [15,16]. Young children with HDM sensitization and allergic diseases require timely diagnosis and early intervention to improve long-term prognosis. Therefore, understanding the clinical characteristics of infants with early HDM sensitization is crucial.

The present study retrospectively analyzed infants who underwent ImmunoCAP testing for HDM, egg white, and cow’s milk sensitization at a pediatric allergy clinic. By comparing the clinical characteristics of HDM-sensitized and non-sensitized infants, this study aimed to elucidate the early clinical significance of HDM sensitization in Korean children and highlight the need for careful monitoring of this high-risk population.

## 2. Materials and Methods

### 2.1. Study Population

This study included infants under 24 months of age who visited the pediatric allergy clinic at Hallym University Hospital Dongtan Sacred Heart Hospital in Korea for evaluation of allergic diseases, including atopic dermatitis, allergic rhinitis, asthma, recurrent wheezing, and food allergies, and who underwent testing for HDM–specific immunoglobulin E (sIgE). Infants in the HDM-positive (HDM [+]) group were defined as those with a *D. farinae* sIgE level ≥ 0.35 kU/L, whereas the HDM-negative (HDM [–]) control group consisted of infants with negative *D. farinae* sIgE results. Controls were individually matched 1:1 to cases. In this study, 1:1 exact matching was performed based on age (in months) and sex to ensure comparability between HDM-sensitized and non-sensitized infants. Exact matching is a statistical technique in which cases and controls are paired based on identical values of selected variables, thereby eliminating confounding from those factors. In our study, only infants sensitized to *D. farinae* were selected, regardless of their sensitization status to *D. pteronyssinus*. *D. farinae* was selected as the representative HDM allergen because it is the earliest and most frequently recognized HDM sensitizer among Korean children [17,18].

### 2.2. Methods

Data were collected retrospectively from electronic medical records. Information on physician-diagnosed allergic diseases was obtained, and results of the ImmunoCAP assay (Thermo Fisher Scientific, Uppsala, Sweden) were analyzed, including sIgE levels to *D. farinae*, egg white, and cow’s milk, as well as total IgE levels. Sensitization to *D. farinae*, egg white, and cow’s milk was defined as sIgE ≥ 0.35 kUA/L, in accordance with the manufacturer’s recommendations and previous clinical guidelines [19,20]. We determined the proportion of infants sensitized to *D. farinae* among all participants under 24 months and among those under 12 months of age. Clinical characteristics and allergic disease patterns were assessed in the HDM (+) group and compared with matched HDM (–) controls using ImmunoCAP results and clinical data.

### 2.3. Statistical Analysis

Categorical variables were summarized as *N* (%), and continuous variables as mean ± standard deviation (SD). Differences between the HDM (+) and HDM (–) groups were evaluated using the Wilcoxon signed-rank test for continuous variables and McNemar’s test for categorical variables. Correlations between immunologic test results were analyzed using Spearman’s rank correlation coefficient. All *p*-values were two-sided, with statistical significance defined as *p* < 0.05. Analyses were performed using R version 4.5.0 (R Foundation for Statistical Computing, Vienna, Austria; https://www.R-project.org/ (accessed on 24 July 2025)).

#### Ethical Considerations

The present study protocol was approved by the Research Ethics Committee of Hallym University Dongtan Sacred Heart Hospital prior to the initiation of the study (approval no. 2021-08-008).

During the preparation of this manuscript, the authors used ChatGPT (version 5o, Open AI) for the purposes of English editing and text polishing. The authors have reviewed and edited the output and take full responsibility for the content of this publication.

## 3. Results

### 3.1. Clinical Characteristics of Infants Sensitized to HDMs

Among 1793 infants under 24 months of age included in the study, 96 (5.4%) demonstrated sensitization to house dust mites (HDMs). Of the 739 infants younger than 12 months, 11 (1.5%) were sensitized, whereas 85 (8.1%) of 1054 children aged 12–24 months showed sensitization. Among infants with HDM sensitization, 74.0% had physician-diagnosed atopic dermatitis and 57.3% had documented food allergies. Additionally, 45.8% experienced recurrent wheezing episodes (≥4) at the time of examination. In a subset analysis of infants under 12 months, 90.9% had atopic dermatitis, all (100%) had food allergies, and 36.4% exhibited recurrent wheezing (Table 1).

### 3.2. Laboratory Findings in HDM-Sensitized Infants

Among HDM-sensitized infants under 24 months of age, 71.9% were co-sensitized to egg white, 56.3% to cow’s milk, and 50% to three or more food allergens. In the subgroup younger than 12 months, the rates of co-sensitization were 90.9% for egg white, 81.8% for cow’s milk, and 72.7% for multiple food allergens (Table 2). Three infants reached the maximum measurable *D. farinae* sIgE level of 100 kU/L. One of these patients, aged 10 months, had a strong family history of allergic diseases (maternal asthma and atopic dermatitis, and allergic rhinitis in both parents) and presented with multiple allergic conditions, including atopic dermatitis, food allergy, infantile asthma, and allergic rhinitis.

Table 1 and Table 2 present descriptive characteristics of HDM-sensitized infants stratified by age (<12 months vs. <24 months). Given the small sample size of the <12 months subgroup (*N* = 11), these data are shown for descriptive purposes only, and no statistical comparisons were performed.

### 3.3. Comparison Between the HDM (+) and HDM (–) Groups

**Figure 1:**
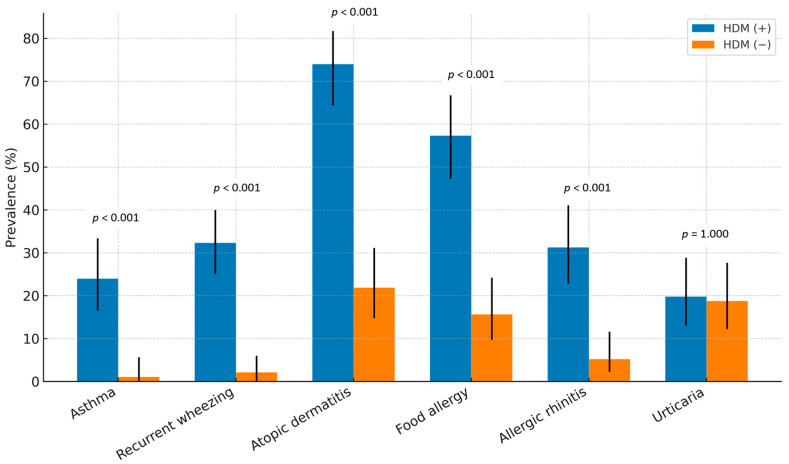
Comparison of clinical characteristics between HDM-sensitized [HDM (+), *N* = 96] and non-sensitized [HDM (−), *N* = 96] infants under 24 months of age. Bars represent prevalence (%), with error bars indicating 95% confidence intervals (CIs).

**Figure 2:**
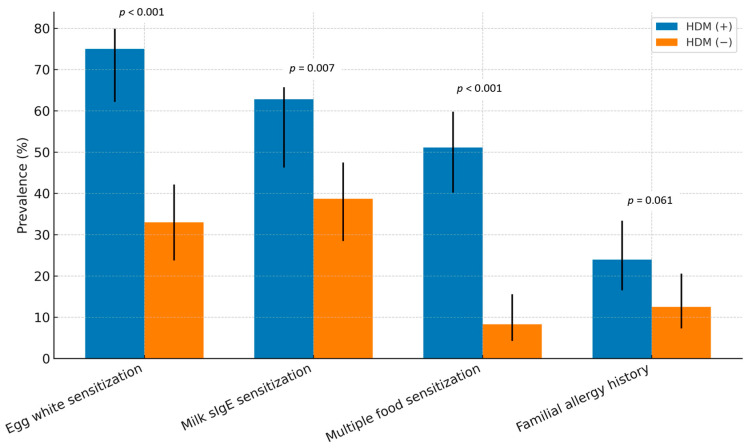
Comparison of sensitization and familial history between HDM-sensitized [HDM (+), *N* = 96] and non-sensitized [HDM (−)] infants under 24 months of age. Bars indicate prevalence (%), with error bars representing 95% confidence intervals.

The prevalence of physician-diagnosed atopic dermatitis (74.0% vs. 21.9%, *p* < 0.001) and physician-diagnosed food allergies (57.3% vs. 15.6%, *p* < 0.001) was significantly higher in the HDM (+) group compared with the HDM (–) group. There was no statistically significant difference in the occurrence of anaphylaxis between the two groups (10.4% vs. 3.1%, *p* = 0.096). Recurrent wheezing (≥4 episodes) (32.3% vs. 2.1%, *p* < 0.001), physician-diagnosed asthma (24.2% vs. 1.0%, *p* < 0.001), and allergic rhinitis (31.2% vs. 5.2%, *p* < 0.001) were significantly more frequent among HDM-sensitized infants, whereas urticaria did not differ between groups. Egg white sensitization was observed in 75.0% of the HDM (+) group compared with 33.0% of the HDM (–) group (*p* < 0.001), and cow’s milk sensitization was also significantly higher (62.8% vs. 38.7%, *p* = 0.007). Multiple food sensitizations (≥3 allergens) were markedly more common in the HDM (+) group (51.1% vs. 8.3%, *p* < 0.001). The food allergens to which the study population was sensitized included egg, cow’s milk, wheat, peanut, soybean, walnut, shrimp, crab, and buckwheat. A positive family history, defined as the presence of at least one allergic disease (asthma, rhinitis, or atopic dermatitis) in either parent, was observed in 23 infants (23.9%) in HDM (+) group and 12 infants (12.5%) in HDM (−) group, with no statistically significant difference between the groups (*p* = 0.062). (Table 3, Figure 1 and Figure 2).

Results of the Wilcoxon signed-rank test indicated significant differences in egg white and cow’s milk sIgE levels between groups. Total IgE values ranged from 8 to over 2500 kU/L and showed a markedly skewed distribution; therefore, values were log-transformed as log(tIgE + 1) to approximate a normal distribution and ensure numerical stability before analysis using paired *t*-tests, which revealed significantly higher total IgE levels in HDM-sensitized infants. Mean egg white sIgE levels were 16.13 kU/L in the HDM (+) group versus 1.15 kU/L in the HDM (–) group, and mean cow’s milk sIgE levels were 6.08 kU/L versus 0.67 kU/L, respectively (Table 4). Within the HDM (+) group, *D. farinae* sIgE levels showed moderate positive correlations with total IgE (*r* = 0.326, *p* = 0.002) and egg white sIgE (*r* = 0.312, *p* = 0.002), and a weak but statistically significant correlation with cow’s milk sIgE (*r* = 0.215, *p* = 0.047) (Table 5).

## 4. Discussion

In this study, the prevalence of HDM sensitization among infants under 2 years of age who visited our hospital’s allergy clinic was 5.4%. Sensitization to HDMs is generally considered uncommon in this age group. However, previous studies suggest marked regional variation. A Belgian study reported that up to 28% of infants under 2 years attending an asthma clinic were sensitized to aeroallergens, predominantly to HDMs [21], and a Taiwanese cohort found sensitization in approximately 30% of children aged 18–24 months, although two-thirds were asymptomatic—a finding attributed to the region’s persistently humid climate [15]. Similarly, in Singapore, HDM sensitization increased from 11% at 18 months to 23% by 3 years [22], while in Shanghai, sensitization rose from <3% in infants under 1 year to >12% by age 3 [23]. In Japan, studies link Der 1 levels in futons and tatami flooring to increased sensitization risk in toddlers [24]. Environmental factors may therefore help explain these differences. In Korea, the traditional floor heating system (ondol) and historically lower use of carpets have been considered protective against HDM exposure. And epidemiological surveys consistently demonstrate that *D. farinae* is the predominant HDM species in Korean children, with higher sensitization rates compared to *D. pteronyssinus* [3,4,10].

Although the relationship between early HDM exposure and subsequent respiratory allergic disease remains a subject of debate [10,25], multiple studies have demonstrated that early HDM sensitization is associated with impaired lung function and adverse respiratory outcomes later in childhood [15,16,26]. In one birth cohort study, even asymptomatic toddlers with early HDM sensitization exhibited higher rates of asthma and allergic rhinitis at 7 years of age, increased exhaled nitric oxide levels, and a greater prevalence of airway hyperresponsiveness [15]. Given the retrospective nature of our study, it was not possible to assess long-term prognoses, such as respiratory disease severity or lung function in infants with early HDM sensitization. Nevertheless, we observed that 32.3% of sensitized infants experienced recurrent wheezing at the time of evaluation, and 22.9% had already been diagnosed with infantile asthma.

In the first year of life, IgE antibody responses are initially directed toward food allergens, followed later by responses to indoor and outdoor aeroallergens [27]. Early onset of sensitization is clinically relevant, as it typically results in a longer duration of sensitization [28,29]. The harmful effects of exposure to indoor allergens, particularly when coupled with allergen sensitization, are most pronounced during the first three years of life [30]. While adaptive immunity reaches maturity after approximately six years of age, the most critical period for establishing mature systemic immune responses occurs between one and two years of age [28,29].

There is increasing evidence that impaired skin barrier function in early life promotes allergen sensitization [31,32]. In infants with atopic dermatitis, genetic factors such as filaggrin (FLG) mutations or inflammation compromise epidermal integrity, allowing allergens to penetrate and skew immune responses toward Th2 pathways with enhanced IgE production [33]. According to the “dual allergen exposure hypothesis,” epicutaneous exposure to food allergens under barrier dysfunction induces sensitization, while oral exposure may promote tolerance [34]. Thus, disrupted skin barriers predispose infants to early food sensitization, which can trigger the “allergic march” and subsequent inhalant allergen sensitization [35]. Food sensitization in infancy has been linked to later sensitization to house dust mites (HDMs) and other inhalant allergens [7]. Consistently, a recent systematic review and meta-analysis demonstrated a dose–response relationship between atopic dermatitis severity and the risk of food allergy, reinforcing the biological plausibility of our observation that HDM-sensitized infants frequently exhibited concomitant atopic dermatitis and egg white sensitization [36]. Moreover, HDM allergens can penetrate damaged skin and induce immune activation, suggesting that barrier dysfunction contributes both to food and direct aeroallergen sensitization [37]. These findings highlight the central role of the skin barrier in shaping immune development and the progression of allergic diseases in childhood.

Sensitization to HDMs via a damaged skin barrier has also been documented. A French study showed that, in infants with atopic dermatitis, sensitization to inhalant allergens occurred later than sensitization to food allergens, and that higher transepidermal water loss was associated with increased rates of inhalant allergen sensitization [38]. In our study, the number of HDM-sensitized infants under 24 months was 96, whereas only 11 infants were under 12 months, limiting the statistical power to detect significant differences between these subgroups. Nevertheless, concomitant atopic dermatitis was observed at a high frequency, affecting 74% of infants under 24 months and 90.9% of those under 12 months. These findings suggest that infants with severe atopic dermatitis may develop aeroallergen sensitization through a compromised skin barrier.

Among infants with HDM sensitization in our cohort, 71.9% were co-sensitized to egg white, 56.3% to cow’s milk, and 50% to three or more food allergens. In those younger than 12 months, the rates were even higher (90.9%, 81.8%, and 72.7%, respectively). Infants sensitized to HDMs also exhibited significantly elevated levels of egg white–specific IgE, cow’s milk–specific IgE, and total IgE compared to non-sensitized infants. These findings suggest that infants with atopic dermatitis who are already sensitized to egg, milk, or multiple foods may be at increased risk for HDM sensitization, warranting clinical evaluation for aeroallergen sensitization in this population.

Previous research has reported a positive correlation between egg white– and HDM-specific immune responses in infants with atopic dermatitis, supporting the hypothesis that food and indoor allergens may concurrently sensitize infants via the skin [39]. A Danish birth cohort study further demonstrated that early-life sensitization to hen’s eggs was associated with asthma and rhinoconjunctivitis at 14 years of age, while transient early HDM sensitization conferred an increased risk of asthma (adjusted odds ratio 3.80) at the same age [40]. In our cohort, infants sensitized to HDMs had a significantly higher prevalence of egg white sensitization compared to those without HDM sensitization, and HDM-specific IgE levels were significantly correlated with egg white–specific IgE levels.

In high-risk infants, HDM sensitization appears to negatively influence respiratory outcomes, emphasizing the need for early environmental interventions aimed at reducing HDM exposure to help prevent sensitization [15,16,26]. Early intervention for HDM sensitization mainly involves environmental control strategies in the sleeping environment, which can reduce allergen exposure but show inconsistent clinical benefits [41,42]. In sensitized children with allergic rhinitis or asthma, sublingual immunotherapy has demonstrated meaningful improvements and potential disease-modifying effects, whereas barrier-focused strategies in early life remain promising but require further HDM-specific evidence [43,44]. However, the pathogenic role of HDM in the atopic march remains speculative, and our interpretation should be considered with caution.

The primary limitation of this study is its retrospective design, which precluded assessment of long-term respiratory outcomes in infants with early HDM sensitization compared to non-sensitized infants. In addition, as this was a single-center analysis conducted in a tertiary pediatric allergy clinic, selection bias may have occurred, and the findings may not be fully generalizable to the broader infant population. Detailed information on environmental HDM exposure, such as indoor humidity, flooring type, and bedding materials, was not available, limiting the ability to directly correlate sensitization with household exposure levels. Furthermore, the relatively small number of infants under 12 months constrained the statistical power of subgroup analyses. Despite these limitations, the use of exact 1:1 matching for age and sex minimized confounding and enhanced the validity of our findings. Future prospective, population-based cohort studies incorporating objective environmental assessments and long-term follow-up will be essential to validate these results and clarify the causal pathways of early HDM sensitization.

## 5. Conclusions

Early-life HDM sensitization in infants was frequently accompanied by multiple food sensitizations, particularly to egg white, and strongly associated with atopic dermatitis. Given these associations, clinicians should consider evaluating HDM sensitization in infants presenting with atopic dermatitis, food allergies, or elevated total and egg white–specific IgE levels to guide early intervention strategies.

## Figures and Tables

**Table 1:** Demographic characteristics of infants with house dust mite sensitization.

	*N* (%) or Mean ± SD	
	Infants < 24 Months (*N* = 96)	Infants < 12 Months (*N* = 11)
Age (months)	17.2 ± 4.3	10.3 ± 0.9
Male	63 (64.6%)	8 (72.7%)
Doctor-diagnosed allergic disease		
Atopic dermatitis	71 (74.0%)	10 (90.9%)
Food allergy	55 (57.3%)	11 (100%)
Anaphylaxis	10 (10.4%)	2 (18.2%)
Asthma	23 (24.2%)	3 (27.3%)
Allergic rhinitis	30 (31.2%)	2 (18.2%)
Recurrent wheezing (≥4 times)	31 (32.3%)	4 (36.4%)

Categorical variables are presented as *N* (%) and continuous variables as mean ± SD.

**Table 2:** Allergy test results of infants with house dust mite sensitization.

Characteristics	*N* (%) or Mean ± SD	
	Infants < 24 Months (*N* = 96)	Infants < 12 Months (*N* = 11)
Total IgE (kU/L)	415.0 ± 674.1	368.2 ± 718.9
HDM sIgE (kU/L)	12.4 ± 23.6	13.3 ± 29.5
Egg white sensitization	69 (75.0%)	10 (90.9%)
Cow’s milk sIgE sensitization	54 (62.8%)	9 (81.8%)
Multiple food sensitization (≥3 food allergens)	48 (51.1%)	8 (72.7%)

HDM, House dust mites; IgE, Immunoglobulin E; sIgE, Specific immunoglobulin E. Categorical variables are presented as *N* (%) and continuous variables as mean ± SD.

**Table 3:** Comparison between the HDM (+) and HDM (−) groups of infants (<24 months).

Variables	*N* (%)		*p*-Value
	HDM (+) Group (*N* = 96)	HDM (−) Group (*N* = 96)	
Physician-diagnosed allergic diseases			
Atopic dermatitis	71 (74.0)	21 (21.9)	<0.001
Food allergy	55 (57.3)	15 (15.6)	<0.001
Anaphylaxis	10 (10.4)	3 (3.1)	0.096
Asthma	23 (24.2)	1 (1.0)	<0.001
Allergic rhinitis	30 (31.2)	5 (5.2)	<0.001
Urticaria	19 (19.8)	18 (18.8)	1.000
Recurrent wheezing (≥4 times)	31 (32.3)	2 (2.1)	<0.001
Family history of allergic diseases	23 (23.9)	12 (12.5)	0.062
Egg white sensitization	69 (75.0)	31 (33.0)	<0.001
Milk sIgE sensitization	54 (62.8)	36 (38.7)	0.007
Multiple food sensitization (≥3 food allergens)	48 (51.1)	8 (8.3)	<0.001

HDM, House dust mites; IgE, Immunoglobulin E; sIgE, Specific immunoglobulin E. HDM (+) versus HDM (–) infants were compared using McNemar’s test.

**Table 4:** Laboratory findings in the HDM (+) and HDM (−) groups of infants (<24 months).

Variables	Mean ± SD		*p*-Value
	HDM (+) Group (*N* = 96)	HDM (−) Group (*N* = 96)	
Log (total IgE + 1)	2.17 ± 0.64	1.57 ± 0.66	≤0.001
Egg white sIgE (kU/L)	16.13 ± 29.65	1.15 ± 3.30	≤0.001
Cow’s milk sIgE (kU/L)	6.08 ± 16.20	0.67 ± 1.36	0.001

HDM, House dust mites; IgE, Immunoglobulin E; sIgE, Specific immunoglobulin E. Values are expressed as mean ± SD. *p*-values for log-transformed total IgE were calculated using the paired *t*-test, whereas *p*-values for the remaining variables were calculated using the Wilcoxon signed-rank test.

**Table 5:** Factors that are correlated with HDM levels.

	Coefficient (*p*-Value)		
	Total IgE	Egg White sIgE	Cow’s Milk sIgE
HDM sIgE	0.326 (0.002)	0.312 (0.002)	0.215 (0.047)

HDM, House dust mites; IgE, Immunoglobulin E; sIgE, Specific immunoglobulin E. Because the variables were not normally distributed, Spearman’s rank correlation test was used to assess relationships between HDM sIgE and total IgE, egg white sIgE, and cow’s milk sIgE. Correlation coefficients (ρ) and corresponding *p*-values are presented in this table.

## Data Availability

The data presented in this study are available on request from the corresponding author due to concerns regarding patient confidentiality.

## Abbreviations

The following abbreviations are used in this manuscript:

HDM	House dust mite
sIgE	Specific immunoglobulin E
